# Theta rhythm-like bidirectional cycling dynamics of living neuronal networks *in vitro*

**DOI:** 10.1371/journal.pone.0192468

**Published:** 2018-02-07

**Authors:** Arseniy Gladkov, Oleg Grinchuk, Yana Pigareva, Irina Mukhina, Victor Kazantsev, Alexey Pimashkin

**Affiliations:** 1 Laboratory of Neuroengineering, Center of Translational Technologies, Lobachevsky State University of Nizhni Novgorod, Nizhny Novgorod, Russia; 2 Cell Technology Department, Central Research Laboratory, Nizhny Novgorod State Medical Academy, Nizhny Novgorod, Russia; 3 Information Science and Technology Department, Skolkovo Institute of Science and Technology, Moscow, Russia; Georgia State University, UNITED STATES

## Abstract

The phenomena of synchronization, rhythmogenesis and coherence observed in brain networks are believed to be a dynamic substrate for cognitive functions such as learning and memory. However, researchers are still debating whether the rhythmic activity emerges from the network morphology that developed during neurogenesis or as a result of neuronal dynamics achieved under certain conditions. In the present study, we observed self-organized spiking activity that converged to long, complex and rhythmically repeated superbursts in neural networks formed by mature hippocampal cultures with a high cellular density. The superburst lasted for tens of seconds and consisted of hundreds of short (50–100 ms) small bursts with a high spiking rate of 139.0 ± 78.6 Hz that is associated with high-frequency oscillations in the hippocampus. In turn, the bursting frequency represents a theta rhythm (11.2 ± 1.5 Hz). The distribution of spikes within the bursts was non-random, representing a set of well-defined spatio-temporal base patterns or motifs. The long superburst was classified into two types. Each type was associated with a unique direction of spike propagation and, hence, was encoded by a binary sequence with random switching between the two “functional” states. The precisely structured bidirectional rhythmic activity that developed in self-organizing cultured networks was quite similar to the activity observed in the *in vivo* experiments.

## Introduction

Synchronization and the interplay between excitation and inhibition in neural networks play crucial roles in the organization of rhythmic activity in the brain [1–5]. Rhythmic oscillatory activity with various frequencies represents a multi-clock substrate for cognitive function, memory and sleep [6, 7]. However, researchers still question whether the rhythmicity emerges from the specific network morphology that develops during neurogenesis [7–11] or it is generated spontaneously due to nonlinear network dynamics mediated by an interplay between excitation and inhibition that is sustained by a homeostatic balance [12–14]. An answer to this fundamental question promises to define the network mechanisms of pathological seizure activity and, hence, to determine treatment approaches. Many brain network functions, normal and pathological states have recently been studied using *in vitro* models [13–23]. In these models, dissociated neuronal cultures provide researchers a unique opportunity to model network dynamics and rhythmicity *in vitro*.

Dissociated neuronal cultures plated on microelectrode arrays after culture for several days *in vitro* (DIV) spontaneously generate activity in the form of periodic and synchronized network burst discharges [24–26, 15]. The bursts display various spatio-temporal distributions of spikes recorded at the electrodes during the discharges. Network bursts may be involved in mechanisms of information encoding [16], memory [17] and chronic neurological diseases, such as epilepsy [18, 27]. For example, similar burst dynamics develop spontaneously or are evoked by a stimulus *in vivo* in the cortex, hippocampus and brain nuclei during brain development [19, 20, 28, 29]. These *in vivo* bursts are associated with a single sharp potential or with spindle-shaped field oscillations (approximately 10 Hz) [19].

Regular bursts in cultured networks are characterized by variable firing rates. Simultaneously, they are composed of highly precise and reproducible spatio-temporal spiking patterns. The spiking patterns are quantified by calculating the values for the timing and the recruitment order of the first spikes initiating the bursts at each electrode, known as activation patterns [30]. These patterns were reported to be stable on a timescale of several hours [30–32]. The profile of the spiking patterns (several tens of milliseconds) at the beginning of the bursts is also precisely repeated in the subsequent bursts, whereas the middle phase of burst formation is highly variable [31]. An analysis of spontaneous activity in cultured networks grown on high-density microelectrode arrays (4096 electrodes) also revealed that only short intervals in the initial parts of the bursts were reproducible and were associated with spike propagation in the network from certain initiation points (neurons) [33]. According to the results of a detailed analysis, bursting activity consists of several motifs that are distinguished by direction or spike propagation pathways and appear randomly during the recording. Several types of patterns, i.e., motifs, have also been observed in the spontaneous bursting activity using activation patterns (only first spike timings) and spiking frequency patterns [21, 34].

The bursting activity in neuronal cultures changes dramatically during development *in vitro* and essentially depends on the initial cell plating density [26, 35]. The minimum plating density of a cortical culture required to produce bursting activity is 250 cells per mm^2^ in Neurobasal medium (neuronal culture medium) [36]. During the first 3 weeks of development, the numbers of GABAergic and glutamatergic terminals increase gradually and simultaneously with the bursting rate [36]. A steady state is reached after 3–4 weeks of culture *in vitro* in hippocampal [37–39] and cortical [36, 40, 41] cultures. In the mature stages of highly dense cultures, the spiking activity consists of complex sequences of a type of burst often called a superburst, with durations ranging from several to tens of seconds. Superbursts are only observed in dense dissociated cultures (2500 rat cortical cells per mm^2^ grown in Dulbecco’s modified Eagle’s medium (DMEM) [22, 26], 4000 cells per mm^2^ grown in Neurobasal medium supplemented with only blockers of inhibitory connections [18] or 8 x 10^3^ and 10^6^ rat hippocampal cells per mm^2^ grown in DMEM [42, 43]). The overall network activity was very complex and characterized by spontaneous superbursts, which, in turn, may cluster into small superburst series [44]. Superbursting activity has also been observed in multilayered neural cultures [45]. High-frequency oscillations resembling network superbursts have been observed even in small but dense neuronal clusters (up to 40 cells) [35]. Numerical simulation of cultured networks revealed that superbursts occurred at earlier stages of network development in larger networks (up to 50 000 neurons) compared to smaller networks (up to 11 000 neurons) [46]. Therefore, the network size is also a crucial parameter [46].

Superbursts in neuronal cultures represent highly coherent spatio-temporal spiking activity patterns that spontaneously develop in originally non-structured networks due to self-organization and plasticity [22].

One of the proposed mechanisms for superbursts is the interaction between the activity of excitatory and inhibitory neurons in culture. In particular, the addition of inhibitory cells from the striatum to the hippocampal cultures was used to study their impact on burst dynamics. An increase in the inhibitory cell fraction in neuronal cultures from 20% to 56% significantly increases the number of small bursts in the superburst structure [47]. The important role of GABAergic neurons in the generation of bursting activity has also been confirmed in network mathematical models in which GABAergic neurons were involved in generating the small bursts in subsequent superburst [46]. Superbursting activity has been increased by treating cultures with blockers of inhibitory synapses (bicuculline and picrotoxin) or a low concentration of Mg^2+^ ions [18, 48] and has been decreased by treating cells with a combination of Na^+^ channel blockers and picrotoxin [18].

The investigation of spiking patterns in the superbursts has revealed remarkably precise repetition of the internal bursting sequence [22]. Superbursts appear with irregular intervals, but their internal structure contains small bursts with highly regular and reproducible activation patterns that persist for hours or days [26]. In another study of cortical cultures during the mature stage (4–6 week *in vitro*), definite motifs observed in the burst activation pattern corresponded to a specific oscillation phase during the ultra-slow oscillations (<0.01 Hz) [12]. These ultra-slow oscillations also represent superbursts with regular intervals. Spiking pattern motifs were found to be strongly conserved across multiple oscillation cycles, repeating themselves with high spatio-temporal precision.

According to one recent mathematical model,high neurite and synapse densities may also influence small bursts in the superburst subsequences [46]. Stable rhythmic activity in the form of propagating synchronized bursts over several minutes was induced in cortical cultures treated with inhibitors of GABAergic synaptic transmission in another study [13]. This periodic synchronized activity on the 3–4 second time scale was only observed at the boundaries of the culture. Thus, the excitatory-inhibitory balance should be an important parameter for generating stable and reproducible synchronized activity in networks.

In the present study, we observed long superbursting activity with well-defined and reproducible temporal dynamics in spontaneously developed hippocampal neurons cultured on a microelectrode array (MEA). Regarding the electrophysiological activity, we observed long (up to 30 seconds) superbursts consisting of subsequences of (up to hundreds of) highly reproducible short bursts in the centre of the cultured network. The spiking frequency in the bursts was 139.0 ± 78.6 Hz, and the interburst interval ranged from 100–150 ms (11.2 ± 1.5 Hz), which resembled unique hippocampal activity under *in vivo* conditions [49]. Spike propagation pathways during short bursts in all long superbursts were aligned along two major spatial directions. The long superburst was encoded into two types, each associated with a definite orientation of spike propagation. The orientation was switched during each single superburst; in the subsequent superburst, the orientation was determined randomly, with a probability of switching to the next orientation of approximately 50%. Therefore, the superburst time sequence was encoded by binary symbols reflecting the spontaneous activation of two dominant spike propagation patterns selected in mature networks of cultured hippocampal cells. Notably, this well-organized rhythmic activity emerged spontaneously in mature culture networks *in vitro* without any specific stimulation and afferentation. We believe that these self-organizing dynamics observed during culture development led to a certain excitatory-inhibitory balance, where cycling dynamics serve as a homeostatic functional state of the culture. Moreover, these findings also suggest a possible mechanism by which different functional states (i.e., vortices and synchronized epileptic-like discharges) may spontaneously appear in brain networks in the absence of stimulation.

## Materials and methods

### Cell culture

**Fig 1:**
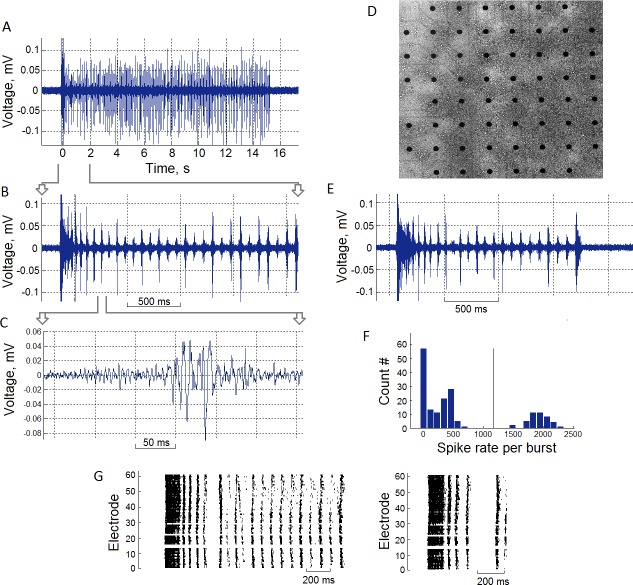
Long superburst activity in hippocampal cultures recorded by the microelectrode array at DIV 35. (A) Electrophysiological signal of spikes within a long superburst recorded from a single electrode. (B) Long superburst at a 2 s timescale and a 200 ms timescale (C). (D) Dissociated hippocampal neurons grown on a microelectrode array (DIV 35). (E) Regular superburst activity with a duration of 1–2 seconds. (F) Initiation burst and subsequent small bursts were separated by clustering the numbers of spikes per burst. The threshold (the vertical line) was identified using K-means clustering. (G) Raster plot of a long superburst (left) and a regular superburst (right).

Hippocampal cells were dissociated from embryonic mice (E18) and plated on microelectrode arrays (MEAs) pre-treated with adhesion promoting molecules of polyethyleneimine (Sigma P3143) with a final density of approximately 15,000–20,000 cells/mm^2^ (Fig 1D). Our cultures were composed of 4–5 layers of the cells. The mice used in our study were received from Institute of Bioorganic Chemistry *Pushchino*, Moscow Region, Russia. C57Bl/6 mice were euthanized via cervical dislocation according to protocols approved by the National Ministry of Public Health for the care and use of laboratory animals. The protocol was approved by the Committee on the Ethics of Animal Experiments of the Nizhny Novgorod State Medical Academy (Permit Number: 9–25.09.2014). All efforts were made to minimize suffering. Embryos were removed and decapitated. The entire hippocampus was dissected under sterile conditions. The cortex, whole medulla and the lower part of the pons were excluded during the dissection. Hippocampi were cut in Ca^2+^- and Mg^2+^-free phosphate-buffered saline (PBS-minus). After enzymatic digestion for 25 min using 0.25% trypsin (Invitrogen 25200–056) at 37°C, cells were separated by trituration (10 passes) using a 1 ml pipette tip. Next, the solution was centrifuged at 1500 g for 1.5 min, and the cell pellet was immediately re-suspended in Neurobasal culture medium (Invitrogen 21103–049) with B27 (Invitrogen 17504–044), glutamine (Invitrogen 25030–024) and 10% fetal calf serum (PanEco К055). The dissociated cells were seeded in a 30 μl droplet covering the centre of the culture dish with 1 mm^2^ electrode region of the MEA. It resulted in a culture 6–7 mm in diameter. After the cells had adhered (usually in 2 hrs), the dishes were filled with 1 ml Neurobasal medium (NBM) supplemented with B-27 and 0.5 mM glutamine with 10% fetal calf serum. After 24 hrs, the plating medium was replaced with a medium containing NBM 2% B-27 and 1% glutamine and 0.5% fetal calf serum but with no antibiotics or antimycotics. Glial growth was not suppressed, given that glial cells are essential for long-term culture health. Half of the medium was replaced every 2 days. The cells were cultured under constant conditions of 35.5°C, 5% CO_2_ and 95% air at saturating humidity in a cell culture incubator (MCO-18AIC, SANYO).

Phase-contrast images of cultures were taken weekly to record the status of the culture using a Leica DMIL HC (Germany) inverted microscope with 10х/0.2 Ph1 objectives. Experiments were conducted when the cultures had been grown for 3–5 weeks *in vitro*.

### Electrophysiological methods

Extracellular potentials were collected using 59 planar TiN electrodes integrated into the USB-MEA-120 system (Multichannel system, Germany). The microelectrode arrays (MEA) had 59 electrodes (8x8 grid) with diameter of 30 μm and spaced 200 μm apart (Fig 1A). Data were recorded simultaneously from 59 channels at a sampling rate of 20 kHz/channel. All signal analysis and statistics were performed using the custom-made software Meaman in Matlab (Mathworks, USA).

### Spike detection

The detection of recorded spikes (Fig 1B) was implemented using threshold calculation:

$$T=N_{S}\sigma ,$$ (1)

where σ = *median* (|*x*| / 0.6745), which was the estimate of the median normalized to standard deviation of a signal with no spikes (see [50] for more details), x is the band-pass-filtered (0.3–8 KHz) signal, and NS is the spike detection coefficient, which was set to 8. The amplitudes of detected spikes were in the range of 20–200 μV. The minimal interspike interval was set to be 1 ms to avoid the overlapping of neighbouring spikes.

### Burst detection

The burst detection method was described in detail in our previous paper [31]. Briefly, we estimated the total spiking rate characteristic, TSR(t), as the number of spikes from all electrodes within each 5 ms time bin. The rapid appearance of a large number of spikes over all electrodes in a small (2 ms) time bin was used as the criterion for burst appearance. Threshold detection was applied to estimate the initiation and termination of the bursts. The burst threshold was set to T_Burst_ = 0.2 × σ_TSR_, where σ_TSR_ is the standard deviation of TSR(t).

The initiation time of the burst was defined as the burst start time, where TSR(t) was greater than the threshold. Next, the initiation time was adjusted to the first spike from all electrodes after a supra-threshold time. Finally, the time point at which TSR crossed the threshold after the burst start time was defined as the burst end time.

**Fig 2:**
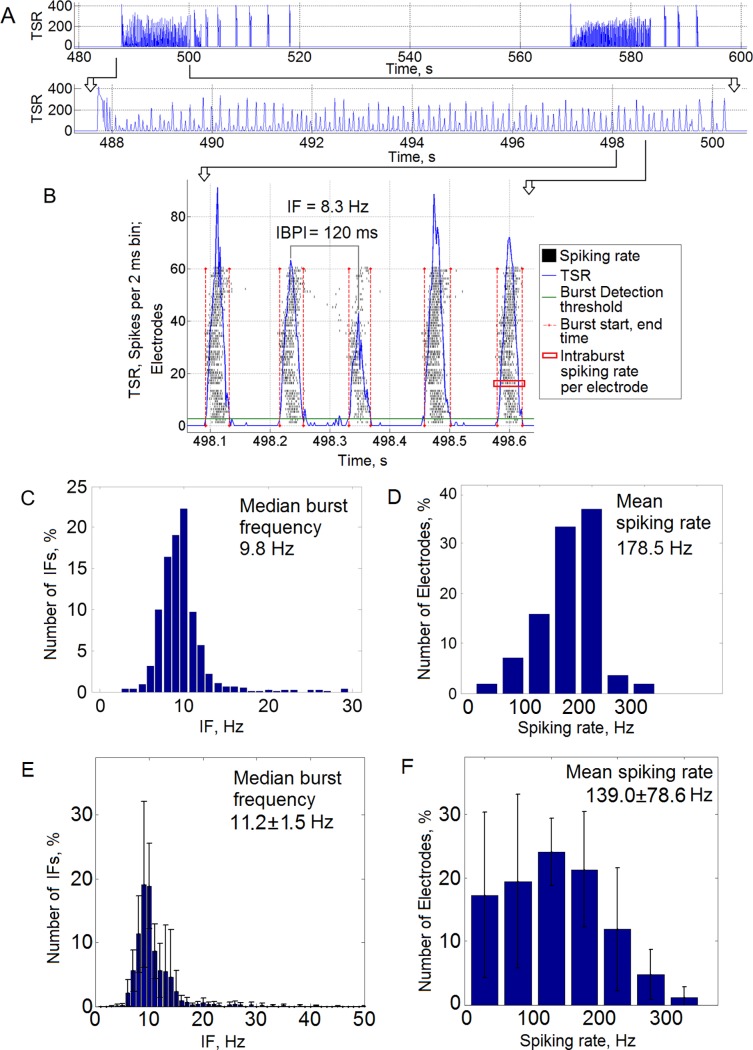
The sequence of small bursts in a long superburst displayed a stable rhythmic structure. (A) Example of the long superburst activity recorded on the MEA, and a fragment of 5 detected small bursts (B). The green horizontal line represents the burst detection threshold, and the red vertical lines represent the burst initiation and end time points. (C) Distribution of burst frequencies (the instantaneous frequency (IF) corresponded to the the interburst peak interval measure (IBPI), see the Methods) in one culture. The median IF (burst frequency) was 9.8 Hz. (D) Distribution of spiking rate frequency from the electrodes with small bursts in one culture. The mean spiking rate was 178.5 Hz. (E) Distribution of IFs (n = 6 cultures). The median burst frequency was 11.2 ± 1.5 Hz (mean ± s.d., n = 6 cultures). (F) Distribution of the spiking rate frequency from the electrodes with small bursts (n = 6 cultures). The spiking rate was 139.0 ± 78.6 Hz (mean±s.d.).

Interburst peak intervals (IBPIs) were calculated as the time interval between adjacent peaks of the spiking rate of detected small bursts. The peaks were separately estimated from the total spike rate (TSR) diagram of each burst. Examples of the peaks and the interval between two bursts are presented in Fig 2B. Such measure of interval between “up” states of the bursts is similar to a measure of oscillation period and frequency in rhythmic activity analysis of *in vivo* recordings. Each IBPI corresponded to the instantaneous frequency (IF) of each pair of the small bursts. All burst pairs were analysed, and IF datasets were used for further statistical analysis.

### Superburst detection

Superbursts and long superbursts in the electrical activity were detected using a previously described method [51]. First, we defined a Gaussian function with an effective width equal to 50 s. Next, that function was iteratively moved from the beginning of the recording to the end using a 10 ms time step, while the cross-correlation of the function with the TSR was calculated for each step. The resulting cross-correlation indicated the amount of synchronized activity (bursts) that was recorded in each 10 s window. We applied a threshold detection algorithm in which the threshold was estimated as the superburst detection accuracy coefficient multiplied by the standard deviation of the calculated cross-correlation to detect superbursts in the spiking activity. The superburst detection accuracy coefficient was estimated empirically and was equal to 0.4. All time points that crossed the threshold were defined as the beginnings and the endings of the superbursts [51].

### Burst classification

Superbursts consisted of initiation bursts lasting for 50–100 ms and short small bursts lasting for 30–50 ms. The total number of spikes within initiation bursts ranged from 1000–3000 spikes, whereas each small burst contained 10–500 spikes (Fig 1F). These two types of bursts were identified using a K-means clustering algorithm.

**Fig 3:**
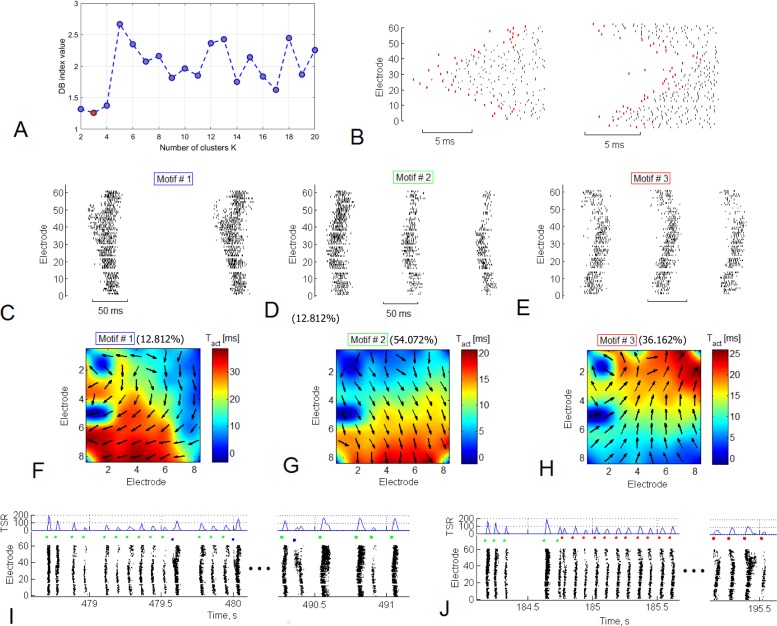
Small bursts in superbursts consist of patterns with bidirectional activity. (A) Small bursts from several long superbursts were clustered into spatio-temporal patterns. The DB index (see the Methods) showed a minimum of 3 clusters, indicating that at most 3 dissimilar groups (motifs) of small bursts are present, according to the activation spiking pattern. (B) Examples of the bursts from two motifs. Red dots indicate the activation pattern—first spike timings for each electrode. Examples of the bursts from 3 motifs (C, D, and E) and spatial representation of *dynamic pattern*s (F, G, and H) are shown. Motif #1 was observed in 10.6% of small bursts, motif #2 was observed in 56.4%, and motif #3 was observed in 32.9% of all small bursts in the superburst. Examples of two types of long superbursts (I and J) that were composed of the 3 motifs. The left long superburst consisted of motif #1 bursts (blue markers) and motif #2 bursts (green markers) (F). The second type of long superburst consisted of bursts of motif #3 (red markers, J).

We analysed activation patterns consisting of first spike timings of the bursts to represent the spatio-temporal properties of all patterns within small bursts. The first spike timing was averaged for each electrode and each small burst. Then, the values from all 60 electrodes were colour coded and plotted on an image using cubic convolution interpolation (Fig 3F, 3G and 3H). This image represented a gradient map of the burst activation profile.

We introduced a vector field map of activation timings in the culture to represent the spatial properties of spike propagation during burst activation. For each MEA electrode, we calculated a vector whose direction represented the activation-timing gradient around an area of 3 electrodes (Fig 3F). Notably, the resulting vector field resembled a colour-coded activation pattern. We defined this spatial representation of the activation pattern as a *dynamic pattern*.

**Fig 4:**
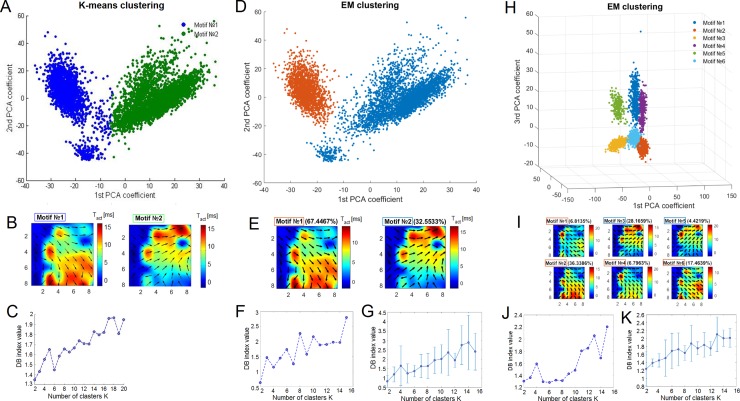
Clustering of the spiking patterns of small bursts in the long superburst. (A) K-means clustering of the activation patterns revealed two motifs (green and blue dots) plotted by principal component analysis (PCA) coefficients. (B) Dynamic patterns represent the average activation patterns of two motifs identified using K-means clustering. The colour represents the average first spike timing of the bursts. (C) Dependence of the DB index on the number of clusters estimated using the K-means analysis. (D) Clustering of the same spiking patterns using EM clustering applied to the two principal components. (E) Dynamic patterns of motifs identified using EM clustering. (F) Dependence of the DB index on the number of clusters estimated using EM clustering and its average (G) (mean±s.d., n = 6 cultures). (H) EM clustering of the spiking patterns applied to the 6 motifs estimated from the three principal components. (I) Dynamic patterns of the 6 motifs estimated by EM using 3 principal components. (J) Dependence of the DB index on the number of clusters estimated using EM clustering with 3 principal components and its average (K) (mean±s.d., n = 6 cultures).

We applied the K-means clustering method to identify motifs of activation patterns in small bursts of all long superbursts. The activation patterns for each small burst consisted of the first spike timings for each of the 59 electrodes of the MEA. This method required a number of clusters to be estimated. First, we estimated two clusters and evaluated cluster separation by calculating the Davies-Bouldin (DB) index [32]. The DB index estimates the ratio between the internal cluster distance and the distance between clusters. Then, the clustering procedure was repeated for various numbers of clusters (2, 3…30), and the DB index was estimated (Fig 4C). The minimum value for the DB index among all tested cluster numbers was 2 or 3, indicating that the activation patterns were optimally clustered into 2 or 3 motifs of small bursts. Small values for the DB index corresponded to compact clusters whose centres were located far from each other. DB values ranging from 0 to 1 indicated “robust” clustering. We also tested the motif separation using the expectation-maximization algorithm (EM clustering). We plotted two principal component coefficients for each activation pattern and highlighted the estimated clusters in different colours to represent the evaluated clusters (Fig 4D). As in the previous case, we used the DB index to evaluate the optimal number of clusters with EM clustering (Fig 4F). This method was applied to 3 principal component coefficients and divided data into the clusters more accurately (Fig 4H, 4I, 4J and 4K), which were used in subsequent analyses.

## Results

First, we analysed the spontaneous activity of the hippocampal cultures. We obtained complex bursting patterns similar to those reported previously in cortical cultures [26]. An example of the spikes recorded from a single electrode within a small burst is shown in Fig 1C. After 3–4 weeks of culture *in vitro*, we obtained the activity described as a superburst (Fig 1E). A typical superburst consisted of a sequence of 3–20 small bursts of 50–100 ms in duration and a 50–150 ms interburst interval. During the period of 30–40 DIV, the cultures generated long superbursts that were similar to regular superbursts, but that lasted for 10–30 seconds and consisted of hundreds of regular small bursts. In summary, we analysed 11 cultures from 3 plating experiments and observed long superbursts in 8 cultures. Six cultures generated more than 6 long superbursts for at least 20 minutes, which were included in the statistical analysis. In other cultures, we observed no more than 2 long superbursts. The signals from a single electrode during the long superburst on timescales of 15 and 2 seconds are illustrated in Fig 1A and 1B. Raster plots of the spiking activity recorded from all 59 electrodes during the superburst and long superburst are shown in Fig 1G. Each point on the raster plot represents the time at which a spike occurred at a particular electrode. The long superbursts were composed of relatively long initiation bursts (50–100 ms) followed by shorter bursts, i.e., the small bursts. The initiation bursts and the small bursts were easily identified by K-means clustering (Fig 1F) using burst firing rate features (see the Methods).

Next, we estimated the frequencies of the small bursts; the initiating bursts and intervals between the long superbursts were excluded from analysis. We estimated instantaneous frequencies of the detected bursts using the interburst peak interval measure (IBPI) (see the Methods) (Fig 2A). Each IBPI corresponded to the instantaneous frequency (IF) of each pair of the small bursts. Notably, the IFs were not normally distributed (Kolmogorov-Smirnov test, p<0.01), and the median value of the IF was estimated for each culture. A typical example of the IF distribution for a small burst sequence in one culture is shown in Fig 2C. Moreover, a median value of the IFs represented a bursting frequency, which was stable and equal to 9.8 Hz. Less than 5% of the IFs ranged from15-30 Hz in presented example. Then, the bursting frequencies were averaged for all cultures, and a mean value was equal to 11.2 ± 1.5 Hz (mean ± standard deviation, n = 6 cultures). An average histogram of the IFs for all 6 cultures is illustrated in Fig 2E demonstrating high reproducibility in various preparations. Most of the IFs were concentrated in the range from 8 to 15 Hz.

We also estimated the mean spiking frequency for each electrode during small bursts exclusively during the intra-burst periods (Fig 2B, the red rectangle marks the period in a sample burst). For the raster plot presented in Fig 1A, the spiking frequency at most electrodes ranged from 100–300 Hz, and the mean frequency was 178.5 Hz (Fig 2D). On average, the spiking frequency per electrode was 139.0 ± 78.6 Hz (mean ± standard deviation, n = 6 cultures, 264 active electrodes) (Fig 2F).

Next, we analysed spiking patterns in sequences of small bursts within a long superburst recorded for 30 minutes. The set of the first spikes in the burst recorded from all electrodes was considered the activation pattern [31]. We applied the EM clustering algorithm for 3 principal component features to investigate different motifs (clusters) of activation patterns. This method required the estimation of a number of clusters. First, using two clusters, we evaluated the cluster separation by calculating the DB index (see the Methods). Then, the clustering procedure was repeated for various numbers of clusters (2, 3…30), and the DB index was estimated (Fig 3A). The minimum value for the DB index among all tested numbers of clusters was 3, indicating that the activation patterns were optimally clustered into 3 motifs in the presented raster plot. The activation patterns and profiles of the spiking patterns in the bursts from separate clusters (motifs) represented different sequences of spike occurrence, i.e., different dynamics of the spike propagation (Fig 3B). Note that the difference in the first spike time sequence in the pattern can be visually observed in Fig 3. Representative raster plots for all small bursts from all 3 motifs are shown in Fig 3C, 3D and 3E. We averaged the activation patterns and calculated the dynamic patterns to investigate the spatio-temporal properties of all patterns within each motif (see the Methods (Fig 3F, 3G and 3H)). The first spike timings from all 59 electrodes were colour coded and plotted on the image created using cubic convolution interpolation. Surprisingly, burst activation was implemented in the form of wave-like spike propagation dynamics with a wide wave front. Arrows represent the gradient of activation times, i.e., the mean direction of spike propagation during burst initiation across each electrode. Eventually, the patterns were organized into a uniform direction in space. Remarkably, motifs #1 and #2 presented similar directions of the activation pattern, from the upper electrodes to the bottom of the MEA, whereas motif #3 presented the opposite direction.

Next, we analysed the sequence in which the motifs appeared in the structure of the long superburst. Some of the long superbursts (Fig 3I) comprised bursts of motifs #1 and #2, whereas other long superbursts in the same recording (Fig 3J) mainly comprised bursts of motif #3.

Notably, the firing rate in the burst sequence (Fig 3J, TSR—total spiking rate) was quite variable, but the sequence of the first spike timings, i.e., the activation patterns, of the bursts remained largely unchanged.

Furthermore, we verified the clustering results for activation patterns using EM clustering for two principal components (PCs) and three PCs and K-means clustering (see the Methods). We applied all three methods to one data set; we varied the number of clusters and estimated clustering using the DB index as shown in Fig 3A. Using K-means and EM clustering with 2 PCs, the DB index had a minimum at 2 clusters (Fig 4C and 4F), whereas the DB index calculated using EM with 3 PCs had a minimum at 6 clusters (Fig 4J). Interestingly, when whole activation patterns (spike timing over 59 electrodes) were reduced to 2 principal components, they were clustered into only two motifs, whereas the patterns reduced to 3 PCs were clustered into 6 motifs. We illustrated all patterns with a colour scale on the 2 PC plots for K-means clustering and EM 2PC clustering (Fig 4A and 4D) and on the 3 PC plot for EM 3PC clustering (Fig 4H) to visualize the clustering results. Even without clustering, the patterns in the 3 PC space were visually identified as the 6 cluster set, whereas K-means and EM 2PCs could only identify 2 clusters. However, we observed the 2 motifs identified using the other algorithms among the 6 motifs (Fig 4B, 4E and 4I). On average, the optimal number of motifs (minimum DB index) calculated using EM clustering for 2 PCs (Fig 4G) and EM for 3 PCs (Fig 4K) displayed a clear minimum value of 2 clusters (n = 6). Interestingly, in all cases, a visual inspection of the clustering identified two major motifs associated with global spike propagation pathways across the MEA. We applied the analysis described below to emphasize this finding.

**Fig 5:**
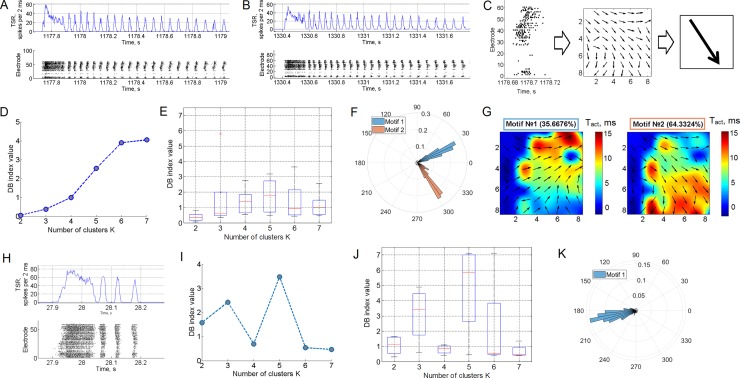
Bidirectional spike propagation pathways form two types of long superbursts. Raster plots of two types (A and B) of the long superburst obtained in one recording (bottom) and its TSR diagram (top, see the Methods). (C) Schematic of major directions in the spike propagation estimation. (D) Dependence of the DB index on the cluster number in the clusterization of major directions in representative raster plots of the long superbursts from 6 cultures (E, boxplot). (F) Histogram of major directions of the bursts from the culture displaying long superbursts. The two coloured clusters in the histogram represent two motifs. (G) Spatial representation of the dynamic patterns of motifs #1 and #2 indicated different spike propagation pathways after clusterization of the major directions. (H) Raster plot of a superburst (bottom) and TSR diagram of the superburst (top, see the Methods). The superburst comprised an initial burst (100–150 ms) followed by a subsequence of 3–10 small bursts. (I) Dependence of the DB index on the cluster number in the clusterization of all major directions of the small bursts in the superburst. (J) Boxplot of the DB index of the small bursts in superbursts (n = 5). (K) Histogram of major directions of the bursts from cultures displaying superbursts.

The activation patterns were characterized by one major direction of a spike propagation pathway. For each pattern, we estimated the angle of the *major direction* by averaging all 59 vectors (Fig 5C). Next, the clustering of the major direction angles revealed two major *direction motifs* in the raster plot (Fig 5A and 5B). The value of the DB index was equal to 0.08 (Fig 5D), which represents two robustly separable clusters, as shown in the histogram of the major direction angles (Fig 5F). Means of the angles from two motifs were equal to 29° and 302°, and the difference was statistically significant (t-test, p<0.01). Interestingly, clustering of the activation patterns composed of spike timings (EM 2 PCs, Fig 4E) and major directions angles showed similar dynamic patterns (Fig 5G and video representation S1 Video). The average DB index of the major direction angle clustering showed that the patterns clearly clustered into two major directions in all cultures (n = 6 cultures) with long superbursts (Fig 5E). The minimum value of the DB index in the cluster estimation was equal to 0.33±0.27 (mean and s.d.). Notably, a DB index with a value less than 1 indicated well-separated clusters when the inter-cluster distance was greater than the intra-cluster volume.

We shifted from analysing long superbursts to analysing regular superbursts for the presence of stable spike propagation pathways to determine whether this form of well-defined bidirectional dynamics is a unique feature of long superbursts. Regular superbursts are also composed of an initial burst (100–150 ms) followed by 3–10 small bursts (Fig 5H). The profile of the small bursts within regular superbursts was less clearly organized than that in the long superburst. In many cases, we were not able to clearly separate the small bursts due to the high variability of activity during development. In this example, 2 clusters could not be estimated correctly because one cluster contained more than 95% of all patterns. Additionally, the DB index was not monotonic (Fig 5I), in contrast to long superburst clustering (Fig 5D), suggesting the absence of the motifs. Indeed, the histogram of all major directions from this raster plot (Fig 5K) indicated the existence of one cluster (e.g., motif) with an average angle of 192°. The characteristics of the DB index averaged over 5 raster plots (n = 5 cultures) did not indicate any clustered structure. Notably, for 2 clusters, the average DB index was equal to 1.06±0.65 (mean and s.d., excluding one sample with <5% in one motif), which was significantly different from the mean DB index for the long superbursts of 0.33±0.27 (n = 6 cultures, t-test, p<0.05). On average, the cluster analysis of the short bursts did not reveal a clear minimum for the DB index in 2 clusters (Fig 5J, boxplot, red lines—median values), in contrast to the recordings containing long superbursts (n = 6 cultures) (Fig 5E). Thus, the patterns in the long superbursts were clustered into two motifs associated with significantly different spike propagation directions, whereas regular superbursts did not show this feature. The mean DB index for the long superbursts 0.33±0.27 was less than 1. These low values indicated the presence of two clusters without inter-cluster overlapping [52] and, hence, are treated as statistical evidence of error-free clustering.

**Fig 6:**
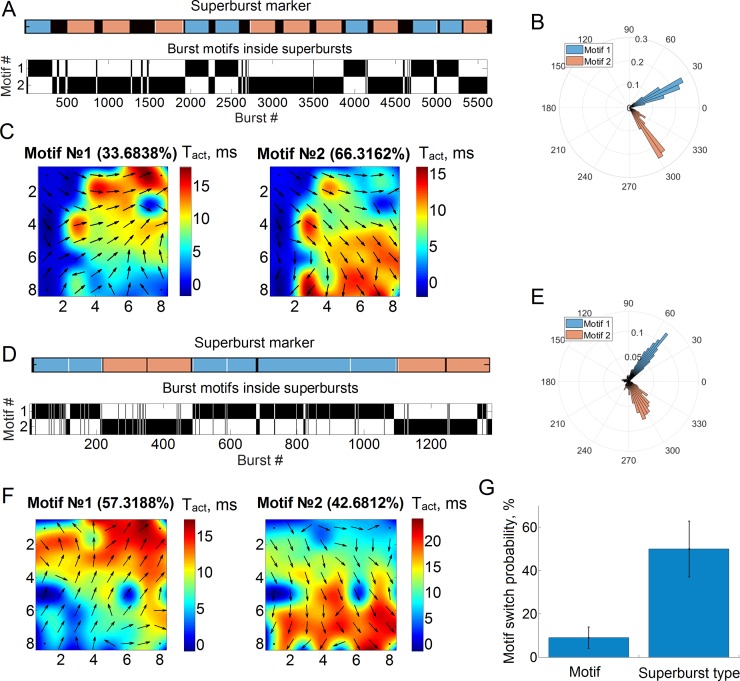
Switch in the spike propagation pathway during long superbursts. (A) Motif appearance within each superburst. Blue and pink bars mark the two types of long superbursts observed in one recording. Black vertical lines illustrate bursts of a particular motif type inside each superburst. Note that one superburst type (blue) was associated with motif #1, and the other (pink) was associated with motif #2. (B) Histogram of major directions for small bursts from cultures displaying long superbursts. The two coloured clusters in the histogram represent the two motifs after clusterization, which appeared in (A). Motif dynamic patterns #1 and #2 are illustrated in C with respect to B. In all cases, the motifs from different types of superbursts have clearly different activation gradients (arrow directions) and major directions (B). The other culture showed similar principal results using the same analysis: two motifs were associated with two types of long superbursts (D), and the bursts from the motifs had significantly different major directions (E, DB index <0.1) and spatio-temporal activation patterns (F). (G) Switch probability of burst patterns during a long superburst (motif) and between subsequent long superbursts (superburst type).

Then, we analysed the reproducibility of motif appearance in a long superburst sequence. Motifs in the long superburst were represented using raster plots (Fig 6A). The vertical black lines in the plot represent the motifs in the sequence of small bursts in all long superbursts. We applied EM clustering to the long superburst based on the motif frequency. This analysis identified two clusters associated with the two types of long superbursts, which are marked in blue and pink in the motif raster plot (Fig 6A, top). Each type appeared randomly in the sequence. Surprisingly, the probability of switching between the two types of long superbursts was 50% ± 13% (n = 6 cultures), indicating the random nature of this activity on a macroscopic timescale. Each long superburst comprised 100–150 small bursts, which were clearly associated with a certain major angle of the motif (Fig 6B). Surprisingly, the first type in the presented example was mostly composed of small bursts of motif #1 (96.9% of all small bursts) and, to a lesser extent, small bursts of motif #2 (3.1%). This finding revealed the presence of stable and directed spatio-temporal patterns of spike propagation pathways during the long superburst activity. In contrast, the other type of long superburst was mostly associated with motif #2 (motif #1–18%, motif #2–82%), representing a different direction of spike propagation. Fig 6C shows the dynamic patterns of the two estimated motifs,in which these major directions are clearly visible. On average, the probability of the appearance of each motif within its own type of the long superburst was equal to 91.5% ± 4.7% (n = 6 cultures). This remarkably high appearance of the motif in the long superbursts clearly indicated the presence of a stable functional structure of the network and, hence, reproducible dynamics.

We also measured the probability of switching between the motif types in the whole raster plot without considering long superburst indexing to quantify the stability of the motifs in the small burst sequences (Fig 6A). The switch probability was quite low, 9% ± 5% (n = 6), indicating that motifs switched quite rarely. Thus, the spontaneous bursting activity employed two basic spike propagation pathways (types of motifs) that were activated and sustained during long superbursts (10–20 sec). Representative images of similar bidirectional activity of the bursts from another culture are shown in Fig 5D, 5E and 5F. Notably, motifs from different long superburst types eventually show significantly different DB index directions of spike pattern propagation using major direction measures or dynamic pattern representations. For each motif, we estimated the mean major angle and the difference between two mean angles. On average, the difference between these angles was 95° ± 31.1° (mean± s.d. n = 6 cultures). Surprisingly, these almost perpendicular spike propagation pathways were spontaneously self-replicated in 6 cultures.

## Discussion

In the present study, the neuronal networks formed by mature hippocampal cultures (30 DIV and older) generated specific network activity with surprisingly long sequences of bursts, i.e., long superbursts of up to hundreds of constituent bursts, with highly regular spiking patterns. In previous studies, superburst activity was reported to display a much shorter duration (up to ten bursts) [26]. In our experiments, we also observed similar activity, but in addition, more than 70% of the cultures (8 of 11) at DIV 30–35 began to spontaneously generate long superbursts with durations of up to hundreds of seconds. Six of 11 cultures generated more than 6 long superbursts during at least 30 minutes. The other cultures generated less than 2 long superbursts with a regular superburst in the background. The precise biophysical mechanism underlying the long superbursts is still largely unknown. We hypothesize that mature cultures (DIV from 35) spontaneously organize into networks with an optimal excitatory-inhibitory balance in which cycling dynamics represent a homeostatic (“natural mode”) pattern that “sustains” the functional connectivity.

This long superbursting activity persisted for several days. Each superburst consisted of an initial burst with the highest firing rate, followed by a subsequence of small bursts with a 50–100 ms duration and a relatively stable 100–200 ms interburst peak interval (IBPI) (Fig 1). This interval corresponded to the bursting frequency 11.2 ± 1.5 Hz (n = 5) (Fig 2), which represented hippocampal rhythmic activity [49]. The observation of this type of periodic bioelectrical activity in a form of theta oscillations in cultures is considered a fundamental feature of hippocampal network formation, which has been widely investigated *in vivo* and in slices *in vitro* [7–11]. In hippocampal neural networks, the theta frequency ranges from 4–10 Hz, and the beta ranges from 10–30 Hz [53, 54]. Other authors define the theta frequency as ranging from 4–12 Hz and beta frequency as ranging from 12–30 Hz [55]. Our analysis indicated a fundamental feature of hippocampal networks that generate theta rhythm, which is involved in behaviour (active motor behaviour [49]), memory tasks and the complex electrophysiological signatures of the theta and beta frequencies during the sleep state [14]. Low-frequency synchronized firing in cortical cultures is associated with classical sleep signatures [14]. The lack of external stimuli during neural network development may trigger stable low-frequency spiking activity in cortical and hippocampal cultures and indicate a functional state of sleep [14].

The spiking rate in the small bursts was 139.0 ± 78.6 Hz (n = 6), which may be associated with high-frequency oscillations and sharp wave-associated ripples in the hippocampus (100–250 Hz) [7, 10] or fast gamma oscillations (90–140 Hz) [56]. A high spiking frequency has also been observed in regular superbursts, and, hence, is not directly associated with the unique characteristics of the observed long superbursts. However, the interplay between the dynamics of small bursts and the generation of high-frequency spiking plays important functional roles *in vivo*. High-frequency oscillations have been found to be modulated by slow theta activity in the isolated rat hippocampus [57] and *in vivo* [58]. Interestingly, coupling of the theta and high-frequency oscillations has been observed during rapid eye movement (REM) sleep, slow-wave sleep and immobility behaviour, whereas ripples are associated with memory consolidation [10].

We postulate that this unique and rare activity appeared in cultures in which a specific balance of morphology and cell density developed spontaneously from the specific initial conditions due to mechanisms of self-organization. The cell density of neurons that survive to DIV 30 dramatically decreased from 2500 cells/mm^2^ to 150 cells/mm^2^ [36]. In another study, the cell density decreased from 5000 cells/mm^2^ to 2500 cells/mm^2^ after two weeks of development in culture [59]. Hippocampal cultures (E18) with a high density of 10^6^ cells/mm^2^ grown in DMEM generate superbursts with a long duration ranging from 46–91 seconds and a relatively low interburst frequency of 0.4–4.6 Hz [43], which is associated with epileptiform activity [60]. Superbursts have also been observed in E17–E18 rat hippocampal cultures with a high density of 7800 cells/mm^2^ [42, 61], but a detailed analysis of activity was not presented. Superbursts with a frequency of 10 Hz were only observed in the presence of increased cAMP concentrations in cultures with a density of 600 cells/mm^2^ [62] or with the addition of dissociated striatal inhibitory cells [47] to cultures with a density of 1000 cells/mm^2^. In our cultures, the initial cellular density was 15000–20000 cells per mm^2^. The plated culture contained approximately 250000 cells and formed 4–5 layers. To our knowledge, our study is the first to record spiking activity under these plating conditions and stage of culture development. Thus, we propose that the plating density of the cultures was the major factor responsible for the appearance of this type of activity.

The culture medium was changed every 2 days in our experiments, whereas in other studies, the medium was changed once or twice a week [15, 26]. Therefore,the frequent changes of small amounts of medium minimized the physiological stress and sustained the homeostatic balance during culture development, which may have affected the stable activity of the cultured network. The densities of glutamatergic and GABAergic synaptic terminals increased during the first 3 weeks *in vitro* and then saturated by DIV 30–35 [63]. In cortical [63] and hippocampal cultures [64–66], the ratio of glutamatergic to GABAergic receptors in synapses and somata during development showed a similar trend to the ratio observed *in vivo*. The ratio of inhibitory and excitatory cells and the cell density present at DIV 30 in our experiments were also important components for the development of the *in vitro* neural networks with such remarkable features of activity and will be investigated in detail in further studies.

Note that we used the culture medium with a low serum concentration (only 0,4%) which is much less than usually used for glial cultures (10%) [67–69]. A neuron/glia ratio decreased after two weeks of culture of hippocampal cells with high density in the conditions of low serum concentration, indicating proliferation of glial cells [39]. The neuron/glia ratio did not change between 2nd and 3rd weeks of the culture [39].The inhibition of astrocyte proliferation can be explained by the effect of accumulated extracellular matrix [51, 70–71] and the contact inhibition of proliferation [72].

Theta rhythmic activity in the hippocampus has been reported to be induced and modulated by external signals originating from the entorhinal cortex. Based on our results, the spiking activity in the range of the hippocampal theta rhythm can be generated in isolated networks of hippocampal cells in the absence of stimulation under stable homeostatic conditions. Further studies using immunohistochemistry will reveal key features of cultures grown under theseconditions.

Importantly, the superbursting activity observed in cortical cultures did not exhibit the long and stable rhythmic activity reported here [14, 22, 26]. The reverberation of activity in the form of periodic synchronized bursts on a time scale of hundreds of milliseconds emerged or was modulated only in response to the suppression of inhibitory synaptic transmission [18, 48]. Superbursting activity in dense cortical cultures (1000–5000 cells/mm^2^) from rats and mice consisted of a sequence of bursts ranging from 0.25 to 1.25 Hz [26,46,73–75]. This activity was associated with epileptic seizures *in vitro* [60]. In rat hippocampal slices, similar epileptiform bursts had a high amplitude (1 mV) and a low-repetition frequency (0.5–1.5 Hz), whereas theta oscillations had a low amplitude (0.5 mV) and a high frequency (5–14 Hz) [76,77]. Moreover, in postnatal cortical neurons cultured at a high density (4000 cells/mm^2^), superbursts were generated with a frequency of 10 Hz in some cases and were induced by inhibitors of GABAergic synaptic transmission [18]. Therefore, the observation of bursting activity in the theta and delta ranges may be unique to the hippocampal cultures.

Notably, the recording area of the MEA (1.6 x 1.6 mm) was located in the centre of the circular culture and had an approximate diameter of 4–5 mm. These spatio-temporal patterns may be part of a global cycling activity with highly stable specific features of the hippocampus *in vivo—*theta rhythmic oscillations. The cycling pattern of the bursting activity might be triggered by pacemaker neurons [78] or may be self-organized in spiral wave dynamics. Similar spike propagation patterns have been observed in cortical cultures with chemically mediated inhibition in the network [13]. Disinhibition of GABAa-mediated synaptic transmission by bicuculline induced episodes of seizures composed of stable, short burst subsequences with 2–3 sec interburst intervals. Further studies using high-density MEA systems or fast CCD cameras for calcium imaging [60] to observe the activity of the whole culture will address these issues.

By analysing the profile of the spiking patterns in the long superbursts, we found that these patterns also become well organized and contain a small number (2–4) of basic motifs, in contrast to the regular superburst activity (Figs 4 and 5). Based on the results obtained from different clustering methods,the patterns of first spike timings in the bursts were segregated into two clusters (motifs).

These motifs defined the presence of two basic types of spike propagation direction in the burst activation pattern. These two “functional” directions further defined the activity in the form of wave-like bidirectional firing patterns that were repeated from burst to burst (Support S1 Video). The stability of the motif appearance within single long superbursts was 91.5% ± 4.7% (n = 6 cultures). This remarkable re-entry of a stable pattern clearly reveals the stable functional structure of the network. Considering the spike timing variability in the culture and clusterization inaccuracy,the motif uniqueness is likely even closer to 100%. Notably, the angle between two major spike propagation pathways of the small bursts’ activation patterns was 95° ± 31.1° (mean± s.d. n = 6 cultures) (Fig 6B and 6E). These almost perpendicular activity propagation pathways were spontaneously self-organized in 6 cultures and were remarkably stable during rhythmic bursting. The long superbursts were also clearly clustered into two types according to the motif appearance in the small burst subsequence (Fig 6A). Each motif appeared mostly within its own type of the long superburst, with a high probability of 91.5% ± 4.7% (n = 6 cultures), indicating that only one of two motifs was generated during each long superburst. We postulate that the dynamics of the neural network were quite stable and reproducible on timescales of milliseconds (activation patterns), seconds (small bursts) and tens of seconds (long superbursts). Interestingly, on a timescale of minutes,in which several long superburst were observed in the activity pattern,the motifs switched from one motif to another with a probability of 50% ± 13% (n = 6 cultures). The switch mainly appeared during the first small burst in the sequence. The first initiation burst with a longer duration and a higher firing rate in the long superburst sequence determined that motif. Therefore, the spiking patterns with unique orientations of activity propagation exhibited an interplay between two complex dynamic states in the network with a stochastic switch on a timescale of several minutes that initiated rhythmic activity with a precise activity pattern on a timescale of seconds and milliseconds. This conclusion complements the results of a regular superburst study [22] and can be further extended to the modelling of brain dynamics during development. Based on our results, hippocampal neuronal cultures display activity with features similar to *in vivo* conditions. Further studies of plating protocols and neuroengineering methods mimicking realistic hippocampal tissue conditions may identify key factors involved in the development of a functional structure in neural networks.

Notably, the well-organized global dynamics of spontaneously developing culture networks are encoded by a binary sequence and are actually represented as a telegraphic signal conveying information about the functional state of the system. We sincerely believe that this stability and reproducibility of the network states will further permit the control of the switching between the states in mature cultures and that these cultures will be useful in the design of living networks with definite functional properties in hybrid information processing systems (neurally controlled robots, “brain-on-chip”, etc.).

## Supporting information

S1 Video Spiking activity patterns in long superbursts. Rasters of two types (motifs) of long superbursts were splitted into time windows of 10 ms duration with 2 ms timestep. The activity within each time window was converted to an image of 8x8 blocks corresponding to MEA electrode mapping. Color of each electrode coded a number of spikes within current time window. First part of the video (A) shows a raster of Fig 5A, second part (B)**–**Fig 5B. One second of the video corresponds to 20 ms of spiking activity.
(MP4)

## References

[1] Vida I, Bartos M, Jonas P. Shunting inhibition improves robustness of gamma oscillations in hippocampal interneuron networks by homogenizing firing rates. Neuron. 2006;49: 107–117. doi: 10.1016/j.neuron.2005.11.036 16387643 10.1016/j.neuron.2005.11.036

[2] Brunel N. What Determines the Frequency of Fast Network Oscillations With Irregular Neural Discharges? I. Synaptic Dynamics and Excitation-Inhibition Balance. J Neurophysiol. 2003;90: 415–430. doi: 10.1152/jn.01095.2002 12611969 10.1152/jn.01095.2002

[3] Mann EO, Paulsen O. Role of GABAergic inhibition in hippocampal network oscillations. Trends Neurosci. 2007;30: 343–349. doi: 10.1016/j.tins.2007.05.003 17532059 10.1016/j.tins.2007.05.003

[4] Haider B. Neocortical Network Activity In Vivo Is Generated through a Dynamic Balance of Excitation and Inhibition. J Neurosci. 2006;26: 4535–4545. doi: 10.1523/JNEUROSCI.5297-05.2006 16641233 10.1523/JNEUROSCI.5297-05.2006 PMC6674060

[5] Isaacson JS, Scanziani M. How inhibition shapes cortical activity. Neuron. Elsevier Inc.; 2011;72: 231–243. doi: 10.1016/j.neuron.2011.09.027 22017986 10.1016/j.neuron.2011.09.027 PMC3236361

[6] Buzsáki G, Watson BO. Brain rhythms and neural syntax: Implications for efficient coding of cognitive content and neuropsychiatric disease. Dialogues Clin Neurosci. 2012;14: 345–367. doi: 10.1097/ALN.0b013e318212ba87 23393413 10.31887/DCNS.2012.14.4/gbuzsaki PMC3553572

[7] Buzsáki G. Rhythms of the Brain. Oxford: Oxford University Press; 2006 doi: 10.1093/acprof:oso/9780195301069.001.0001

[8] Buzsáki G. Theta oscillations in the hippocampus. Neuron. 2002;33: 325–340. doi: 10.1016/S0896-6273(02)00586-X 11832222 10.1016/s0896-6273(02)00586-x

[9] Goutagny R, Jackson J, Williams S. Self-generated theta oscillations in the hippocampus. Nat Neurosci. 2009;12: 1491–3. doi: 10.1038/nn.2440 19881503 10.1038/nn.2440

[10] Tort ABL, Scheffer-Teixeira R, Souza BC, Draguhn A, Brankák J. Theta-associated high-frequency oscillations (110-160Hz) in the hippocampus and neocortex. Prog Neurobiol. 2013;100: 1–14. doi: 10.1016/j.pneurobio.2012.09.002 23022096 10.1016/j.pneurobio.2012.09.002

[11] Pavlov I, Savtchenko LP, Song I, Koo J, Pimashkin A, Rusakov D a, et al Tonic GABAA conductance bidirectionally controls interneuron firing pattern and synchronization in the CA3 hippocampal network. Proc Natl Acad Sci U S A. 2014;111: 504–9. doi: 10.1073/pnas.1308388110 24344272 10.1073/pnas.1308388110 PMC3890854

[12] Mok SY, Nadasdy Z, Lim YM, Goh SY. Ultra-slow oscillations in cortical networks *in vitro*. Neuroscience. 2012;206: 17–24. doi: 10.1016/j.neuroscience.2012.01.009 22266346 10.1016/j.neuroscience.2012.01.009

[13] Keren H, Marom S. Long-range synchrony and emergence of reentry in neural networks. Sci Rep. Nature Publishing Group; 2016;6: 1–17. doi: 10.1038/s41598-016-0001-8 27874019 10.1038/srep36837 PMC5118796

[14] Colombi I, Tinarelli F, Pasquale V, Tucci V, Chiappalone M. Corrigendum: A simplified *in vitro* experimental model encompasses the essential features of sleep [Front. Neurosci., 10, (2016), (315)] doi: 10.3389/fnins.2016.00315 Front Neurosci. 2016;10. doi:10.3389/fnins.2016.00409 10.3389/fnins.2016.00315 PMC4935686 27458335

[15] Chiappalone M, Koudelka-hep M. Network dynamics and synchronous activity in cultured cortical neurons. Int J Neural Syst. 2007;17: 87–103. doi: 10.1142/S0129065707000968 17565505 10.1142/S0129065707000968

[16] Krahe R, Gabbiani F. Burst firing in sensory systems. Nat Rev Neurosci. 2004;5: 13–23. doi: 10.1038/nrn1296 14661065 10.1038/nrn1296

[17] Huerta PT, Lisman JE. Bidirectional synaptic plasticity induced by a single burst during cholinergic theta oscillation in CA1 *in vitro*. Neuron. 1995;15: 1053–63. http://dx.doi.org/10.1016/0896-6273(95)90094-2 7576649 10.1016/0896-6273(95)90094-2

[18] Kim JH, Heo R, Choi JH, Lee KJ. Dynamic transitions among multiple oscillators of synchronized bursts in cultured neural networks. J Stat Mech Theory Exp. 2014;4: P04019 doi: 10.1088/1742-5468/2014/04/P04019

[19] Khazipov R, Sirota A, Leinekugel X, Holmes GL, Ben-Ari Y, Buzsáki G. Early motor activity drives spindle bursts in the developing somatosensory cortex. Nature. 2004;432: 758–761. doi: 10.1038/nature03132 15592414 10.1038/nature03132

[20] Leinekugel X, Khazipov R, Cannon R, Hirase H, Ben-Ari Y, Buzsáki G. Correlated bursts of activity in the neonatal hippocampus in vivo. Science. 2002;296: 2049–2052. doi: 10.1126/science.1071111 12065842 10.1126/science.1071111

[21] Madhavan R, Chao ZC, Potter SM. Plasticity of recurring spatiotemporal activity patterns in cortical networks. Phys Biol. 2008;4: 181–193. doi: 10.1088/1478-3975/4/3/005.Plasticity 17928657 10.1088/1478-3975/4/3/005 PMC2577584

[22] Wagenaar DA, Nadasdy Z, Potter SM. Persistent dynamic attractors in activity patterns of cultured neuronal networks. Phys Rev E—Stat Nonlinear, Soft Matter Phys. 2006;73: 1–17. doi: 10.1103/PhysRevE.73.051907 16802967 10.1103/PhysRevE.73.051907 PMC2570189

[23] Leondopulos SS, Boehler MD, Wheeler BC, Brewer GJ. Chronic stimulation of cultured neuronal networks boosts low frequency oscillatory activity at theta and gamma with spikes phase-locked to gamma frequencies. j Neural Eng. 2012;9: 1–20. doi: 10.1088/1741-2560/9/2/026015.Chronic 22361724 10.1088/1741-2560/9/2/026015 PMC3376752

[24] Maeda E, Robinson HP, Kawana A. The mechanisms of generation and propagation of synchronized bursting in developing networks of cortical neurons. J Neurosci. 1995;15: 6834–6845. Available: http://www.jneurosci.org/content/15/10/6834 7472441 10.1523/JNEUROSCI.15-10-06834.1995 PMC6578010

[25] Maeda E, Kuroda Y, Robinson HP, Kawana a. Modification of parallel activity elicited by propagating bursts in developing networks of rat cortical neurones. Eur J Neurosci. 1998;10: 488–96. Available: http://www.ncbi.nlm.nih.gov/pubmed/9749711 9749711 10.1046/j.1460-9568.1998.00062.x

[26] Wagenaar D a, Pine J, Potter SM. An extremely rich repertoire of bursting patterns during the development of cortical cultures. BMC Neurosci. 2006;7: 11 doi: 10.1186/1471-2202-7-11 16464257 10.1186/1471-2202-7-11 PMC1420316

[27] McCormick D, Contreras D. On the cellular and network bases of epileptic seizures. Annu Rev Physiol. 2001;63: 815–846. doi: 10.1146/annurev.physiol.63.1.815 11181977 10.1146/annurev.physiol.63.1.815

[28] Chiu C, Weliky M. Spontaneous activity in developing ferret visual cortex in vivo. J Neurosci. 2001;21: 8906–8914. Available: http://www.jneurosci.org/content/21/22/8906.long 11698602 10.1523/JNEUROSCI.21-22-08906.2001 PMC6762264

[29] Weliky M, Katz LC. Correlational structure of spontaneous neuronal activity in the developing lateral geniculate nucleus in vivo. Science. 1999;285: 599–604. doi: 10.1126/science.285.5427.599 10417392 10.1126/science.285.5427.599

[30] Shahaf G, Eytan D, Gal A, Kermany E, Lyakhov V, Zrenner C, et al Order-based representation in random networks of cortical neurons. PLoS Comput Biol. 2008;4: e1000228 doi: 10.1371/journal.pcbi.1000228 19023409 10.1371/journal.pcbi.1000228 PMC2580731

[31] Pimashkin A, Kastalskiy I, Simonov A, Koryagina E, Mukhina I, Kazantsev V. Spiking signatures of spontaneous activity bursts in hippocampal cultures. Front Comput Neurosci. 2011;5: 46 doi: 10.3389/fncom.2011.00046 22087091 10.3389/fncom.2011.00046 PMC3213406

[32] Pimashkin A, Gladkov A, Agrba E, Mukhina I, Kazantsev V. Selectivity of stimulus induced responses in cultured hippocampal networks on microelectrode arrays. Cogn Neurodyn. Springer Netherlands; 2016; 1–13. doi: 10.1007/s11571-016-9380-6 27468317 10.1007/s11571-016-9380-6 PMC4947052

[33] Gandolfo M, Maccione A, Tedesco M, Martinoia S, Berdondini L. Tracking burst patterns in hippocampal cultures with high-density CMOS-MEAs. J Neural Eng. 2010;7: 056001 doi: 10.1088/1741-2560/7/5/056001 20720282 10.1088/1741-2560/7/5/056001

[34] Raichman N, Ben-Jacob E. Identifying repeating motifs in the activation of synchronized bursts in cultured neuronal networks. J Neurosci Methods. 2008;170: 96–110. doi: 10.1016/j.jneumeth.2007.12.020 18281097 10.1016/j.jneumeth.2007.12.020

[35] Idelson MS, Ben-Jacob E, Hanein Y. Innate Synchronous Oscillations in Freely-Organized Small Neuronal Circuits. PLoS One. 2010;5: 1–9. doi: 10.1371/journal.pone.0014443 21203438 10.1371/journal.pone.0014443 PMC3010988

[36] Ito D, Tamate H, Nagayama M, Uchida T, Kudoh SN, Gohara K. Minimum neuron density for synchronized bursts in a rat cortical culture on multi-electrode arrays. Neuroscience. Elsevier Inc.; 2010;171: 50–61. doi: 10.1016/j.neuroscience.2010.08.038 20800660 10.1016/j.neuroscience.2010.08.038

[37] Ide N, Andruska A, Boehler M, Wheeler BC, Brewer GJ. Chronic network stimulation enhances evoked action potentials. J Neural Eng. 2010;7: 1–15. doi: 10.1088/1741-2560/7/1/016008 20083862 10.1088/1741-2560/7/1/016008 PMC3775841

[38] Koryagina EA, Pimashkin AS, Kazantsev VB, Mukhina IV. Dynamics of stimulated bioelectrical activity in neural networks *in vitro*. Vestn Lobachevsky Univ Nizhni Novgorod. 2011;2: 254–261.

[39] Shirokova OM, Frumkina LE, Vedunova MV, Mitroshina EV, Zakharov YN, Khaspekov LG, et al Morphofunctional Patterns of Neuronal Network Developing in Dissociated Hippocampal Cell Cultures. Sovrem Tehnol v Med. 2013;5: 6–12.

[40] Chiappalone M, Bove M, Vato A, Tedesco M, Martinoia S. Dissociated cortical networks show spontaneously correlated activity patterns during *in vitro* development. Brain Res. 2006;1093: 41–53. doi: 10.1016/j.brainres.2006.03.049 16712817 10.1016/j.brainres.2006.03.049

[41] Van Pelt J, Corner M a., Wolters PS, Rutten WLC, Ramakers GJ a. Longterm stability and developmental changes in spontaneous network burst firing patterns in dissociated rat cerebral cortex cell cultures on multielectrode arrays. Neurosci Lett. 2004;361: 86–89. doi: 10.1016/j.neulet.2003.12.062 15135900 10.1016/j.neulet.2003.12.062

[42] Tokuda M, Kiyohara A, Taguch T, Kudoh SN. The effects of the current stimulation on electrical activity in dissociated neurons. 20th Anniversary MHS 2009 and Micro-Nano Global COE—2009 International Symposium on Micro-NanoMechatronics and Human Science. 2009. pp. 118–122. 10.1109/MHS.2009.5352068

[43] Zhu G, Li X, Pu J, Chen W, Luo Q. Transient alterations in slow oscillations of hippocampal networks by low-frequency stimulations on multi-electrode arrays. Biomed Microdevices. 2010;12: 153–158. doi: 10.1007/s10544-009-9370-0 19937128 10.1007/s10544-009-9370-0

[44] Gladkov AA, Pimashkin AS, Lepina AP, Kazantsev VB, Mukhina IV. Features of neural network response caused by electrical stimulation in mature hippocampal cell culture of mice. Vestn Lobachevsky state Univ Nizhni Novgorod. 2014;1: 57–64. Available: http://www.vestnik.unn.ru/en/nomera?anum_eng=8248

[45] Frega M, Tedesco M, Massobrio P, Pesce M, Martinoia S. Network dynamics of 3D engineered neuronal cultures: a new experimental model for in-vitro electrophysiology. Sci Rep. 2014;4: 5489 doi: 10.1038/srep05489 24976386 10.1038/srep05489 PMC4074835

[46] Gritsun TA, le Feber J, Rutten WLC. Growth Dynamics Explain the Development of Spatiotemporal Burst Activity of Young Cultured Neuronal Networks in Detail. PLoS One. 2012;7 doi: 10.1371/journal.pone.0043352 23028450 10.1371/journal.pone.0043352 PMC3447003

[47] Chen X, Dzakpasu R. Observed network dynamics from altering the balance between excitatory and inhibitory neurons in cultured networks. Phys Rev E—Stat Nonlinear, Soft Matter Phys. 2010;82: 1–8. doi: 10.1103/PhysRevE.82.031907 21230108 10.1103/PhysRevE.82.031907

[48] Huang YT, Cheung YL, Song H, Lai PY, Chan CK. 8th Int. Meeting on Substrate-Integrated Microelectrode Arrays. Spontaneous reverberation in developing neuronal culture networks. Reutlingen, Germany; 2012. pp. 86–87.

[49] Robinson E. Hippocampal rhythmic slow activity (RSA, theta): A critical analysis of selected studies and discussion of possible species-differences. Brain Res. 1980;2: 69–101. http://dx.doi.org/10.1016/0165-0173(80)90004-1 10.1016/0165-0173(80)90004-1 6772282

[50] Quiroga RQ, Nadasdy Z, Ben-Shaul Y. Unsupervised spike detection and sorting with wavelets and superparamagnetic clustering. Neural Comput. 2004;16: 1661–87. doi: 10.1162/089976604774201631 15228749 10.1162/089976604774201631

[51] Vedunova M, Sakharnova T, Mitroshina E, Perminova M, Pimashkin A, Zakharov Y, et al Seizure-like activity in hyaluronidase-treated dissociated hippocampal cultures. Front Cell Neurosci. 2013;7: 149 doi: 10.3389/fncel.2013.00149 24062641 10.3389/fncel.2013.00149 PMC3770920

[52] Davies DL, Bouldin DW. A cluster separation measure. IEEE Trans Pattern Anal Mach Intell. 1979;1: 224–227. doi: 10.1109/TPAMI.1979.4766909 21868852

[53] Penttonen M, Buzsáki G. Natural logarithmic relationship between brain oscillators. Thalamus Relat Syst. 2003;2: 145–152. doi: 10.1016/S1472-9288(03)00007-4

[54] Sirota A, Csicsvari J, Buhl D, Buzsáki G. Communication between neocortex and hippocampus during sleep in rodents. Proc Natl Acad Sci U S A. 2003;100: 2065–2069. doi: 10.1073/pnas.0437938100 12576550 10.1073/pnas.0437938100 PMC149959

[55] Kopell N, Ermentrout GB, Whittington M a, Traub RD. Gamma rhythms and beta rhythms have different synchronization properties. Proc Natl Acad Sci U S A. 2000;97: 1867–1872. doi: 10.1073/pnas.97.4.1867 10677548 10.1073/pnas.97.4.1867 PMC26528

[56] Sullivan D, Csicsvari J, Mizuseki K, Montgomery S, Diba K, Buzsáki G. Relationships between hippocampal sharp waves, ripples, and fast gamma oscillation: influence of dentate and entorhinal cortical activity. J Neurosci. 2011;31: 8605–8616. doi: 10.1523/JNEUROSCI.0294-11.2011 21653864 10.1523/JNEUROSCI.0294-11.2011 PMC3134187

[57] Jackson J, Goutagny R, Williams S. Fast and slow gamma rhythms are intrinsically and independently generated in the subiculum. J Neurosci. 2011;31: 12104–12117. doi: 10.1523/JNEUROSCI.1370-11.2011 21865453 10.1523/JNEUROSCI.1370-11.2011 PMC6623234

[58] Soltesz I, Deschenes M. Low- and high-frequency membrane potential oscillations during theta activity in CA1 and CA3 pyramidal neurons of the rat hippocampus under ketamine-xylazine anesthesia. J Neurophysiol. 1993;70: 97–116. Available: http://jn.physiology.org/content/70/1/97 doi: 10.1152/jn.1993.70.1.97 8395591 10.1152/jn.1993.70.1.97

[59] Le Feber J, Stoyanova II, Chiappalone M. Connectivity, excitability and activity patterns in neuronal networks. Phys Biol. IOP Publishing; 2014;11 doi: 10.1088/1478-3975/11/3/036005 24828208 10.1088/1478-3975/11/3/036005

[60] Bao W, Wu J-Y. Propagating wave and irregular dynamics: spatiotemporal patterns of cholinergic theta oscillations in neocortex *in vitro*. J Neurophysiol. 2003;90: 333–341. doi: 10.1152/jn.00715.2002 12612003 10.1152/jn.00715.2002 PMC2941800

[61] Kiyohara A, Taguchi T, Kudoh SN. Effects of electrical stimulation on autonomous electrical activity in a cultured rat hippocampal neuronal network. EEJ Trans Electr Electron Eng. 2011;18: 163–167. doi: 10.1002/tee.20639

[62] Niedringhaus M, Chen X, Dzakpasu R, Conant K. MMPs and soluble ICAM-5 increase neuronal excitability within *in vitro* networks of hippocampal neurons. PLoS One. 2012;7: 1–9. doi: 10.1371/journal.pone.0042631 22912716 10.1371/journal.pone.0042631 PMC3418258

[63] Ito D, Komatsub T, Goharab K. Measurement of saturation processes in glutamatergic and GABAergic synapse densities during long-term development of cultured rat cortical networks. Brain Res. 2013;1534: 22–32. Available: http://doi.org/10.1016/j.brainres.2013.08.004 doi: 10.1016/j.brainres.2013.08.004 23948099 10.1016/j.brainres.2013.08.004

[64] Benson DL, Cohen P a. Activity-independent segregation of excitatory and inhibitory synaptic terminals in cultured hippocampal neurons. J Neurosci. 1996;16: 6424–32. Available: http://www.ncbi.nlm.nih.gov/pubmed/8815921 8815921 10.1523/JNEUROSCI.16-20-06424.1996 PMC6578921

[65] Benson DL, Tanaka H. N-Cadherin Redistribution during Synaptogenesis in Hippocampal Neurons. J Neurosci. 1998;18: 6892–6904. Available: http://www.jneurosci.org/content/18/17/6892.long 9712659 10.1523/JNEUROSCI.18-17-06892.1998 PMC6792987

[66] Yamada MK, Nakanishi K, Ohba S, Nakamura T, Ikegaya Y, Nishiyama N, et al Brain-derived neurotrophic factor promotes the maturation of GABAergic mechanisms in cultured hippocampal neurons. J Neurosci. 2002;22: 7580–5. 22/17/7580 [pii] 12196581 10.1523/JNEUROSCI.22-17-07580.2002 PMC6757965

[67] De Lima AD, Voigt T. Astroglia inhibit the proliferation of neocortical cells and prevent the generation of small GABAergic neurons *in vitro*. Eur J Neurosci. 1999;11: 3845–3856. doi: 10.1046/j.1460-9568.1999.00804.x 10583473 10.1046/j.1460-9568.1999.00804.x

[68] Zhao X, Wang XW, Zhou KS, Nan W, Guo YQ, Kou JL, et al Expression of Ski and its role in astrocyte proliferation and migration. Neuroscience. 2017;362: 1–12. doi: 10.1016/j.neuroscience.2017.08.027 28844002 10.1016/j.neuroscience.2017.08.027

[69] Ferrer-Acosta Y, Gonzalez-Vega MN, Rivera-Aponte DE, Martinez-Jimenez SM, Martins AH. Monitoring astrocyte reactivity and proliferation *in vitro* under ischemic-like conditions. JVisExp. 2017; 21;(128): 1–9. doi: 10.3791/55108 29155711 10.3791/55108 PMC5755171

[70] Krushel LA, Tai MH, Cunningham BA, Edelman GM, Crossin KL. Neural cell adhesion molecule (N-CAM) domains and intracellular signaling pathways involved in the inhibition of astrocyte proliferation. Proc Natl Acad Sci U S A. 1998;95: 2592–6. doi: 10.1073/pnas.95.5.2592 9482931 10.1073/pnas.95.5.2592 PMC19425

[71] Struve J, Maher PC, Li YQ, Kinney S, Fehlings MG, Kuntz IVC, et al Disruption of the hyaluronan-based extracellular matrix in spinal cord promotes astrocyte proliferation. Glia. 2005;52: 16–24. doi: 10.1002/glia.20215 15892130 10.1002/glia.20215

[72] Nakatsuji Y & Miller RH. Homotypic cell contact-dependent inhibition of astrocyte proliferation. Glia. 1998; 22; 4: 379–389. doi: 10.1002/(SICI)1098-1136(199804)22:4<379::AID-GLIA7>3.0.CO;2-Z 9517570 10.1002/(sici)1098-1136(199804)22:4<379::aid-glia7>3.0.co;2-z

[73] Reimer T, Baumann W, Gimsa J. Population bursts of parvalbumin-positive interneurons inhibit spiking pyramidal cells in spontaneously active cortical. 2012;6: 1033–1042. Available: http://www.davidpublishing.com/davidpublishing/Upfile/1/11/2013/2013011102854784.pdf

[74] Stephens CL, Toda H, Palmer TD, Demarse TB, Ormerod BK. Adult neural progenitor cells reactivate superbursting in mature neural networks. Exp Neurol. Elsevier Inc.; 2012;234: 20–30. doi: 10.1016/j.expneurol.2011.12.009 22198136 10.1016/j.expneurol.2011.12.009

[75] Wagenaar DA. Development and control of epileptiform bursting in dissociated cortical cultures. 2006. Dissertation (Ph.D.), California Institute of Technology. Available: http://resolver.caltech.edu/CaltechETD:etd-07032005-170918

[76] Konopacki J, Kowalczyk T, Golebiewski H, Eckersdorf B, Konopacki J. Window effect of temperature on carbachol- induced theta-like activity recorded in hippocampal formation *in vitro*. Brain Res. 2001;901: 184–194. doi: 10.1016/S0006-8993(01)02355-1 11368966 10.1016/s0006-8993(01)02355-1

[77] Konopacki J, Gdqbiewski H, Eckersdorf B. *In vitro* recorded theta-like activity in the limbic cortex: Comparison with spontaneous theta and epileptiform discharges. Acta Neurobiol Exp (Wars). 2000;60: 67–85. Available: http://www.ane.pl/linkout.php?vol=60&no=1&fpp=67 10769932 10.55782/ane-2000-1327

[78] Gritsun TA, Le Feber J, Stegenga J, Rutten WLC. Network bursts in cortical cultures are best simulated using pacemaker neurons and adaptive synapses. Biol Cybern. Springer Berlin / Heidelberg; 2010;102: 293–310. doi: 10.1007/s00422-010-0366-x 20157725 10.1007/s00422-010-0366-x

